# Gonadotropin-Inhibitory Hormone Plays Roles in Stress-Induced Reproductive Dysfunction

**DOI:** 10.3389/fendo.2017.00062

**Published:** 2017-04-05

**Authors:** Takeshi Iwasa, Toshiya Matsuzaki, Kiyohito Yano, Minoru Irahara

**Affiliations:** ^1^Department of Obstetrics and Gynecology, Institute of Biomedical Sciences, Tokushima University Graduate School, Tokushima, Japan

**Keywords:** gonadotropin-inhibitory hormone, RFRP-3, Rfrp, stress, hypothalamic–pituitary–gonadal, sexual behavior

## Abstract

Physical and psychological stressors suppress hypothalamic–pituitary–gonadal axis activity and sexual behavior and consequently induce reproductive dysfunction. Recently, it has been shown that gonadotropin-inhibitory hormone (GnIH), also called RFamide-related peptide 3 (RFRP) in mammals, which is a potent inhibitory regulator of gonadotropin-releasing hormone (GnRH) and gonadotropin, is involved in stress-induced reproductive dysfunction. GnIH/Rfrp (the gene coding RFRP-3) expression and activity are increased by psychological and immune stress, and this alteration suppresses GnRH and gonadotropin secretion. Glucocorticoid acts as a mediator that interacts between stress and hypothalamic GnIH/RFRP-3. GnIH/RFRP-3 also plays important roles in stress-induced suppression of sexual behavior and infertility, and genetic silencing of GnIH/Rfrp completely recovers sexual behavior and fertility. This review summarizes what is currently known about the roles of GnIH in stress-induced reproductive dysfunction.

## Introduction

Humans and animals have a finite amount of energy for their activities. Therefore, if any activity has to be energetically prioritized, energy for other activities will be suppressed. Although these changes may play some role in regulation of homeostasis, they occasionally result in negative consequences for normal physiological function and accelerate some diseases. Reproductive functions are often suppressed when large amounts of energy will be used for other physiological functions because such reproductive processes are not essential for individual survival (1). Several kinds of stress, such as infection, psychological burden, and excess of exercise, are thought to be pivotal triggers of reproductive dysfunctions in humans and animals (2–4). Generally, stress activates some endocrine and immune systems, such as the hypothalamic–pituitary–adrenal axis and pro-inflammatory cytokines, to regulate homeostasis. However, such alterations act to suppress reproductive function at the same time.

Reproductive function is mainly regulated by the hypothalamic–pituitary–gonadal (HPG) axis in humans and animals. Physical and psychological stressors suppress HPG activity through inhibition of gonadotropin-releasing hormone (GnRH) in both males and females (5–7), thereby decreasing luteinizing hormone (LH) and follicle-stimulating hormone release from the pituitary (8, 9). When the relationships between stress and reproductive functions are evaluated experimentally, inflammatory stress induced by a Gram-negative bacterial cell wall component, lipopolysaccharide (LPS), and psychological stress induced by restraint stress are frequently used. Similar to other stressors, these stresses induce some sickness behaviors, and they also suppress HPG activity through inhibition of GnRH synthesis and secretion in several mammals and birds (10–17). In addition, it has been well established that the actions of some stress-related endocrine, neuroendocrine, and inflammatory factors, such as pro-inflammatory cytokines, corticotropin-releasing hormone, and glucocorticoid/corticosterone, are increased by stress and that these alterations act to decrease GnRH and gonadotropin secretion in times of stress (15, 18). However, the results of studies have been slightly controversial, and it has been assumed that some other factors may also be involved in the stress-induced suppression of HPG activity. A breakthrough occurred in the early 21st century when a novel RFamide peptide that directly suppresses GnRH/gonadotropin synthesis and secretion was newly discovered (19). In birds, this neuropeptide was named gonadotropin-inhibitory hormone (GnIH), and later studies have shown that GnIH and its receptor, G-protein-coupled receptor (GPR) 147, have pivotal roles in the regulation of physiological function of the HPG axis, such as GnRH pulses and surges, in many species (20–22). The mammalian orthologous gene and peptide for avian GnIH are called Rfrp and RFRP-3, respectively. It has been gradually shown that GnIH/RFRP-3 and GPR147 also play important roles in the stress-induced suppression of reproductive dysfunction.

This paper presents a review of what is currently known about the roles of GnIH/RFRP-3 in stress-induced reproductive dysfunction in experimental animals. The main focus is on the relationship between GnIH/RFRP-3 and the HPG axis, but the role of GnIH/RFRP-3 in reproductive behavior under stress is also examined.

## The Role of GnIH/RFRP-3 in Stress-Induced Suppression of the HPG Axis

**Figure 1:**
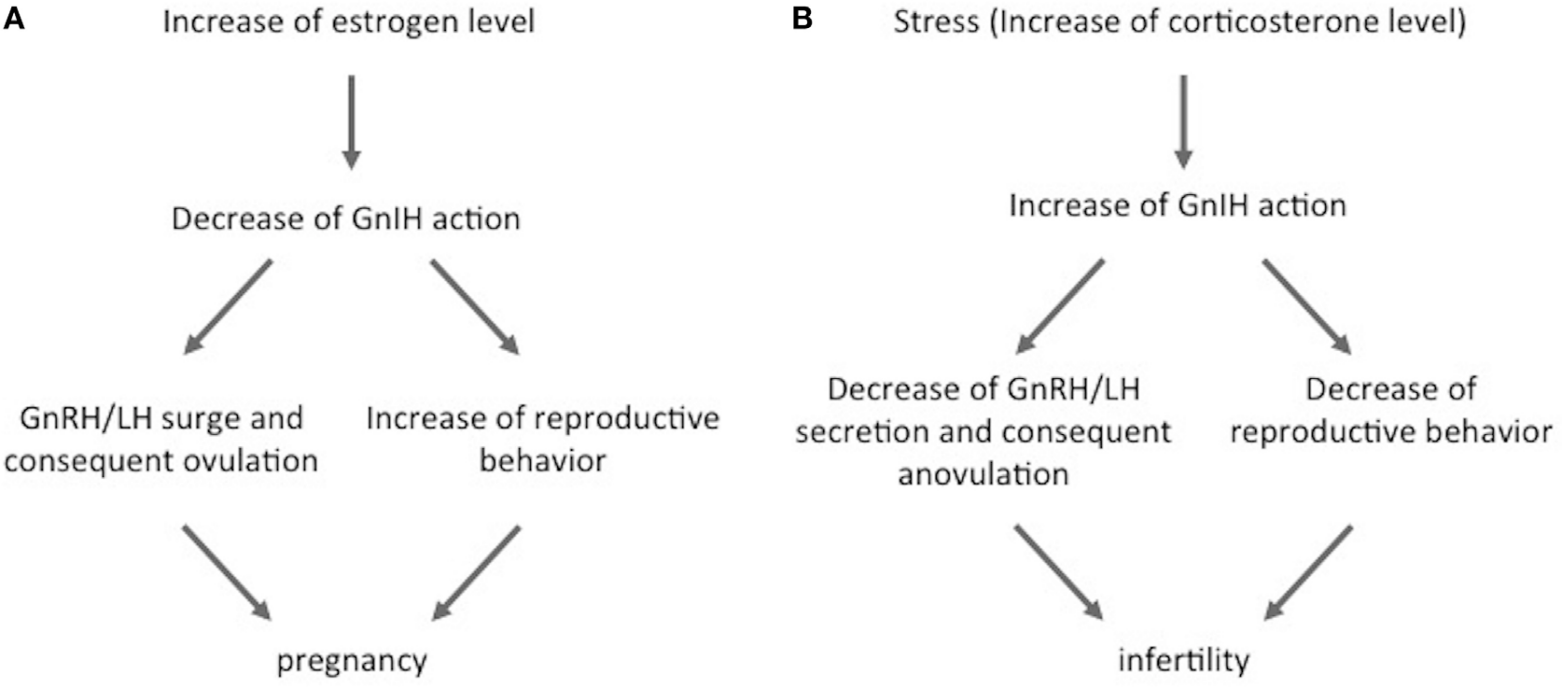
**The role of gonadotropin-inhibitory hormone (GnIH) under physiological and stress conditions**. **(A)** GnIH participates in the control of physiological GnRH/luteinizing hormone (LH) regulation. GnIH activity is decreased by high estrogen concentrations at the time of the GnRH/LH surge. This alteration promotes both ovulation and reproductive behaviors and consequently increases the chance of pregnancy. **(B)** On the other hand, GnIH plays roles in stress-induced reproductive dysfunction. GnIH expression and activity are increased by stress, and this alteration suppresses the hypothalamic–pituitary–gonadal axis action by inhibition of GnRH/LH secretion. It also decreases reproductive behavior and, consequently, decreases the chances of pregnancy and induces infertility. Such stress-induced changes of GnIH may be partially induced by corticosterone secreted from the adrenal gland.

In the year 2000, Tsutsui and colleagues discovered a novel neuropeptide that actively suppresses gonadotropin release from cultured bird pituitary (19). Because this was the first demonstration of a hypothalamic factor that suppresses gonadotropin release, this neuropeptide was named GnIH based on its biological action. Thereafter, GnIHs were further identified in other vertebrates, mammals, primates, and humans (21–23). Because the identified neuropeptides possess LPXRFamide (X = L or Q) motif at their C-termini, the mammalian GnIH orthologous gene and peptide are called Rfrp and RFRP-3, respectively. GnIH/RFRP-3 neurons project to the median eminence in birds and female sheep and suppress the secretion and synthesis of gonadotropin *via* GnIH/RFRP-3 receptor GPR147 under both *in vivo* and *in vitro* conditions in male birds, female rats, and female sheep (19, 22, 24, 25). GnIH/RFRP-3 neurons also project to GnRH neurons and inhibit their activity *via* GPR147 in birds and mammals (22, 26, 27). Therefore, GnIH/RFRP-3 inhibits gonadotropin secretion and synthesis through direct and indirect actions on the pituitary. It has been shown that GnIH/RFRP-3 inhibits GnRH-elicited gonadotropin release and decreases LH pulse amplitude in female sheep (22, 24), and GnIH/RFRP-3 activity is decreased by high estradiol (E2) concentrations at the time of the GnRH/LH surge in female hamsters (Figure 1A) (20, 22). Similarly, elevated estrogen lowers Rfrp mRNA levels in male and female mice (28). These results show that GnIH/RFRP-3 plays important roles in the regulation of the HPG axis to maintain normal reproductive ability. On the other hand, it has recently been shown that some kinds of stresses induce acute and chronic elevations of the number of GnIH/RFRP-3-immunoreactive cells and GnIH gene expression in the hypothalamus and that these changes disrupt the function of the HPG axis and, consequently, suppress reproductive ability. Kirby et al. found that acute (3 h, measured immediately after stress) and chronic (14 days, 3 h/day, measured 24 h after the end of the last stress) immobilization stresses, common psychological stress models, lead to upregulation of Rfrp mRNA expression levels in the dorsomedial hypothalamic area, and that changes of Rfrp mRNA levels correlate negatively with serum LH levels in male rats (29). In addition, they showed that 53% of RFRP-3 neurons express glucocorticoid receptor (GR) and that adrenalectomy abolishes the chronic immobilization stress-induced increase in Rfrp expression levels (29). Similarly, corticosterone administration increases Rfrp mRNA expression levels *in vitro* in rHypoE23, which is an Rfrp-expressing cell line derived from rat hypothalamus (30). These effects of corticosterone on rHypoE-23 are blocked by a GR antagonist (31, 32). Treatment with cortisol in fish increases GnIH mRNA levels and reduces GnRH mRNA and serum LH levels (33). In addition, dexamethasone exposure during the neonatal period in female mice increases Rfrp mRNA levels, and it reduces GnRH mRNA levels and delays pubertal onset (34). These data indicate that hypothalamic GnIH/RFRP-3 integrates the suppressive effects of glucocorticoid on the HPG axis under psychological stress conditions. Recently, Peragine et al. showed that RFRP-3 suppresses sexual maturation in socially non-dominant female rats living in colonies, where breeding is monopolized by dominant animals (35). This result also indicates that RFRP-3 may be involved in the social stress-induced suppression of reproductive function.

Recently, we evaluated the relationship between immune stress and hypothalamic Rfrp mRNA expression levels in female rats. An injection of a septic dose (5 mg/kg) of LPS in female rats was found to increase hypothalamic Rfrp and GPR147 mRNA levels 6 h after injection, whereas it reduced serum LH levels and hypothalamic GnRH mRNA levels (36). In this condition, Rfrp mRNA levels were negatively correlated with GnRH mRNA and serum LH levels. Interestingly, a lower dose (500 µg/kg) of LPS did not change Rfrp and GPR147 mRNA levels, although it decreased serum LH levels. Similarly, Lopes et al. have shown in birds that an injection of 2 mg/kg of LPS suppressed GnRH mRNA and peptide expressions 2 h after injection, but did not affect GnIH (37). These results suggest that the underlying mechanisms of dysfunction of gonadotropin secretion are changed according to the severity of immune stress, and that changes of some reserve factors, i.e., GnIH/RFRP-3, begin to participate in the suppression of GnRH and gonadotropin under severe conditions. On the other hand, the factors involved in the upregulation of Rfrp in times of immune stress have not been elucidated. Although we think that not only glucocorticoid but also pro-inflammatory cytokines play some roles, further examinations are needed to clarify this hypothesis. It has also been reported that metabolic challenge, a type of energetic stress, has no effect on Rfrp mRNA levels or RFRP-3 neuronal activity in female mice (38). This result indicates that GnIH/RFRP-3 mediates some kinds of, but not all, stressors.

In summary, GnIH/RFRP-3 plays roles in the suppression of the HPG axis in times of stress (Figure 1B). Glucocorticoid may be one of the mediators that transmit the stress signal to hypothalamic GnIH/RFRP-3 neurons. However, it is also possible that the importance of GnIH/RFRP-3 in HPG dysfunction may be changed according to the kind and severity of stressor.

## The Role of GnIH/RFRP-3 in Stress-Induced Suppression of Reproductive Behaviors

As well its role in the HPG axis, GnIH/RFRP-3 also plays some roles in the regulation of reproductive behavior in some species. Johnson et al. found that central injection of RFRP-3 decreased sexual behavior in male rats (39), whereas Piekarski et al. reported that it decreased sexual motivation without affecting lordosis behavior in female hamsters (23, 40). Piekarski et al. also showed that administration of RFRP-3 affected neuronal activity in some hypothalamic nuclei, i.e., preoptic area, medial amygdala, and the bed nucleus of the stria terminalis, which are implicated in female sexual behaviors. Therefore, it had been assumed that a stress-induced increase of GnIH/RFRP-3 activity might suppress not only the HPG axis but it may also suppress sexual behavior and, consequently, promote infertility or subfertility. Recently, Geraghty et al. published an excellent report on this matter. In their study, they showed that chronic (3 h/day for 18 days) immobilization stress in female rats led to elevated hypothalamic Rfrp mRNA expression levels both immediately after and 4 days after the end of stress (41). This chronic stress did not affect the estrous cycle, but it decreased sexual behavior and pregnancy rates and increased embryo resorption when they mated 4 days after cessation of stress. The authors further showed that genetic silencing of Rfrp with shRNA during stress completely recovered the sexual behavior, pregnancy rate, and litter size when the females were mated after cessation of stress. These results indicate that stress-induced GnIH/RFRP-3 is related to dysfunction of sexual behavior under stress, including effects on the pregnancy rate and litter size (Figure 1B). Therefore, GnIH/RFRP-3 may be one of the clinical targets to prevent stress-induced infertility.

## Conclusion

Recent studies have shown that GnIH/RFRP-3 plays roles in stress-induced reproductive dysfunction in many species. A stress-induced increase of GnIH/RFRP-3 actions not only suppresses the HPG axis but also disrupts sexual behavior, and these alterations have adverse effects on the pregnancy rate and litter size. These results indicate that GnIH/RFRP-3 may be one of the clinical targets to restore stress-induced infertility. However, because there are only limited data about the roles of GnIH/RFRP-3 in humans, more evaluations in humans would be needed to apply GnIH/RFRP-3 in a clinical setting.

## Author Contributions

All authors listed have made substantial, direct, and intellectual contributions to the work and approved it for publication.

## Conflict of Interest Statement

The authors declare that the research was conducted in the absence of any commercial or financial relationships that could be construed as a potential conflict of interest.
